# Cystinuria-Related Urinary Stone as the Cause of Repeated Urinary Tract Infections (UTIs) in a Child

**DOI:** 10.7759/cureus.78003

**Published:** 2025-01-26

**Authors:** Anastasios Panagiotis Chantzaras, Spyridon Karageorgos, Anna Papakonstantinou, Christos Kyriopoulos, Panagiotis Mitsos

**Affiliations:** 1 Department of Pediatric Respiratory Medicine, Evelina London Children's Hospital, London, GBR; 2 First Department of Pediatrics, Agia Sofia Children's Hospital, Athens, GRC; 3 Department of Urology, Agia Sofia Children's Hospital, Athens, GRC

**Keywords:** congenital anomaly of the kidneys and the urinary tract, interest in pediatric urology, medicine-pediatrics, pediatric hospital medicine, renal stone surgery, urinary tract infection

## Abstract

Urinary tract infections (UTIs) are one of the most common bacterial infections in children and are associated with both acute and long-term complications. Most UTIs typically resolve after antibiotic treatment. However, UTIs may be the initial clinical manifestation of a potential congenital anomaly of the kidneys and the urinary tract (CAKUT) or may indicate the presence of a renal stone. Especially in pediatric patients presenting with early and recurrent episodes of UTIs, a thorough clinical and radiographical evaluation is warranted.

Our manuscript presents a case of a four-year-old male patient diagnosed with a 4.4 cm-diameter stone in his bladder. The patient was investigated for recurrent *Enterococcus faecalis* lower UTIs and was finally diagnosed with cystinuria-related urolithiasis.

## Introduction

Urinary tract infections (UTIs) are one of the most common bacterial infections in pediatric patients and are associated with both acute and long-term complications. These include but are not limited to systemic hypertension, renal scarring, and chronic renal failure [1]. The prevalence of UTIs in childhood varies according to sex and age. Boys have a higher incidence of UTIs during the first six months of life (5.3%), which subsequently decreases to approximately 2% at the age of six years old. On the other hand, UTIs are less common in girls during the first six months of life (2%), while incidence increases up to 11% at the age of six years old [2].

Most pediatric UTIs are caused by fecal flora Gram-negative bacteria that typically ascend the urinary tract, with *Escherichia coli* (*E. coli*) being the main causative agent. In addition, *Klebsiella*, *Proteus*, *Enterobacter*, and *Enterococcus *spp. are also common pathogens [3]. Although UTIs typically resolve with antibiotic treatment, they could be the initial clinical manifestation of an underlying congenital anomaly of the kidneys and the urinary tract (CAKUT) [4]. The isolation of non-*E. coli* bacteria, such as *Enterococcus* spp., is highly indicative of such abnormalities, a finding that clinicians should bear in mind when determining which cases require further diagnostic investigations with imaging studies [5]. For instance, vesicoureteral reflux (VUR) is revealed in 30% of children after the first episode of UTI [6]. The presence of VUR increases the risk of recurrent UTIs, while bladder and bowel dysfunction and innate immunity defects are being recently appreciated as risk factors as well [7].

Here, we present a case of a four-year-old male patient with recurrent *Enterococcus faecalis* (*E. faecalis*) UTI on the background of cystinuria urolithiasis.

## Case presentation

A four-year-old boy presented to the pediatric emergency department with a three-day history of dysuria without fever. His medical background was remarkable for two episodes of *E. faecalis*-related UTIs over the last six months, which were effectively treated with oral antibiotics as an outpatient. Family history was free of chronic medical diseases.

On examination, the patient was growing and developing well, and his body weight was 17 kg. The abdomen was soft and non-tender on palpation; no organomegaly or any palpable abdominal mass was noted. Respiratory and cardiovascular examinations were normal, without peripheral edema. Vital signs were within normal values, and blood pressure was measured at 88/60 mmHg. Blood tests revealed normal renal function and negative inflammatory markers, while the urine dipstick was indicative of UTI with the presence of pyuria.

**Figure 1:**
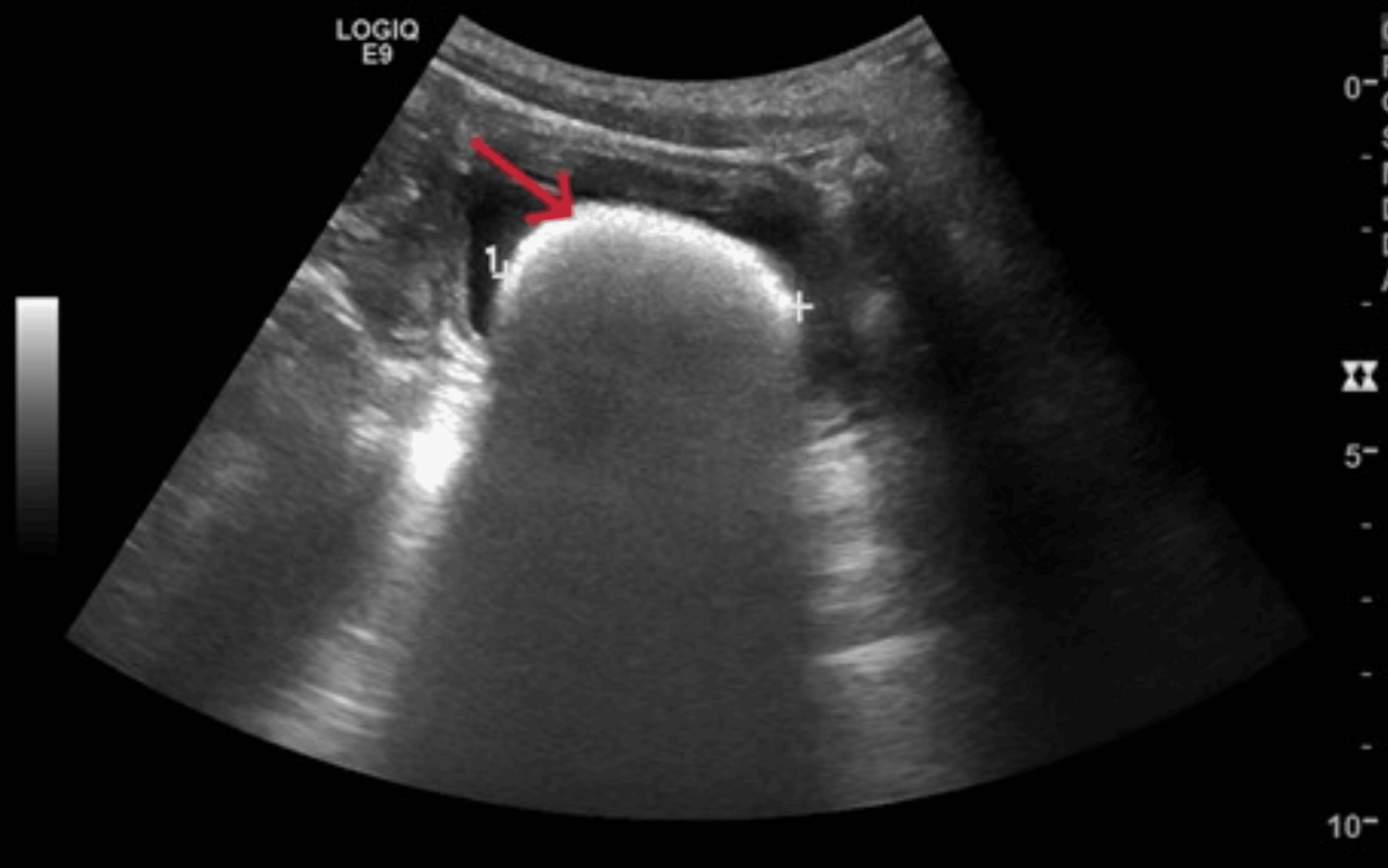
A renal ultrasound reveals a radiopaque stone in the patient’s bladder. The red arrow highlights the stone and the cross highlights the diameter of the stone.

A urine culture was sent, which substantiated the third episode of *E. faecalis* UTI in six months. Due to recurrent episodes of non-*E. coli* UTIs, renal ultrasound was performed, depicting a 4.4 cm-diameter hyper-reflective stone in the patient’s bladder (Figure 1)

Further investigation of the etiology of our patient’s urolithiasis was requested, and we performed a 24-hour urine collection. Results showed normal calcium, oxalate, and uric acid levels, whereas there was increased cystine excretion, indicative of cystinuria; cystine was measured at 323 mg (reference range: 7-11 mg).

**Figure 2:**
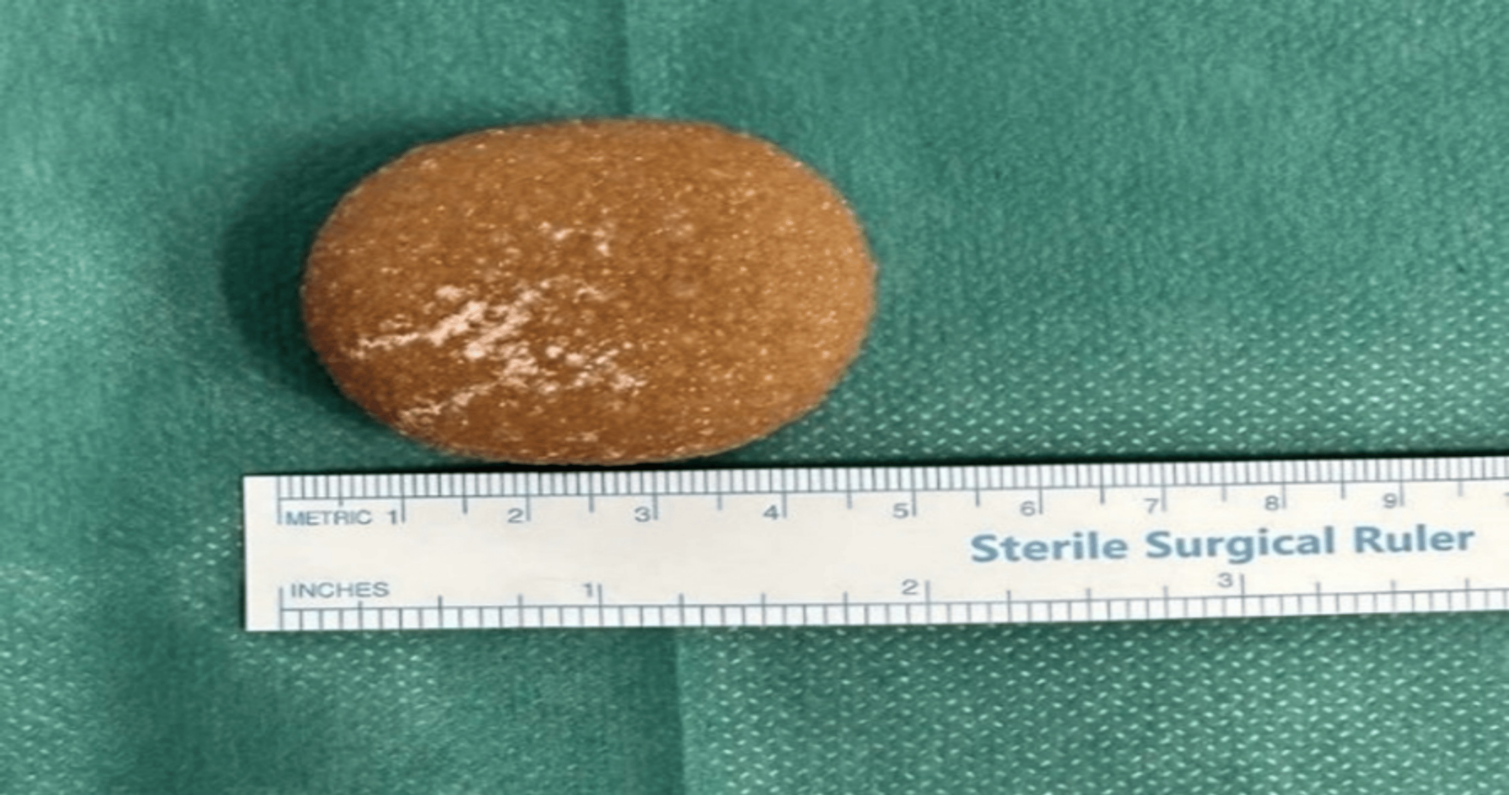
A 4.4 cm-diameter cystine stone was retrieved via cystolithotomy. Chemical analysis of the stone confirmed the diagnosis of cystinuria.

Given the increased size of the stone, urologic consultation was requested, and the patient underwent cystolithotomy for stone removal. The stone was retrieved (Figure 2) and sent for analysis, in which cystine crystals were revealed and the conclusive diagnosis of cystinuria was reached.

The patient remained afebrile and asymptomatic post surgery and was discharged after two days with diet modifications and sodium bicarbonate to prevent stone recurrence. On his six-month follow-up appointment, our patient was asymptomatic and compliant with therapeutic and non-therapeutic interventions.

## Discussion

In our work, we present a preschool-age boy with recurrent atypical UTIs whose diagnostic workup led to the diagnosis of cystinuria urolithiasis.

A recent retrospective study including 7,730 children who presented to primary care facilities or emergency departments with a first episode of atypical UTI failed to report a higher risk of structural renal abnormalities in the non-*E. coli* UTI patients group compared to the group with *E. coli* UTI patients [8]. However, the National Institute for Health and Care Excellence (NICE) Guidelines for the diagnosis and management of UTIs in the pediatric population report that further imaging investigation is warranted in children presenting with an episode of atypical UTI or recurrent UTIs to exclude underlying abnormalities [3]. In this vein, our patient, who had a history of both recurrent and non-*E. coli* UTIs, an ultrasound was performed, which revealed urolithiasis.

Urolithiasis is a condition caused by the formation of stones in the urinary tract. Based on the site of the stones, it is further divided into nephrolithiasis (kidney stones), ureterolithiasis (ureteral stones), and cystolithiasis (bladder stones). The incidence of urolithiasis in the pediatric population has increased to approximately 10% over the past 20 years, which is attributed to living conditions, including dietary and climate changes [9]. Regarding stone composition, 70% to 80% consist of calcium oxalate, 10 to 15% of struvite, 10% of calcium phosphate, and <5% of uric acid. Cystine stones represent 6% to 8% of all pediatric urinary stones [10].

Cystinuria is the most common inherited cause of urolithiasis in the pediatric population, and most patients present with the first episode of UTI in the first decade of life. It is an autosomal recessive disease caused by mutations in two genes: the SLC3A1 gene on chromosome 2p16.3 and the SLC7A9 gene on chromosome 19q13.1 [11]. Based on the affected gene, the disease is classified in Type A (SLC3A1 gene), Type B (SLC7A9 gene), and Type AB (both genes), with the prevalence reported as 45%, 53%, and 2%, respectively [12]. Patients have impaired cystine absorption in the renal proximal tubules, leading to elevated cystine excretion in the urine and cystine calculi formation due to the low solubility of the cystine at normal urine pH [11].

Stone analysis remains the gold standard for determining the composition of the urinary stone and establishing the diagnosis of cystinuria. Nevertheless, a useful non-invasive method for determining urine cystine hyperexcretion is the measurement of cystine in 24-hour urine collection. Healthy subjects typically have <30 mg of daily cystine urine excretion, whereas cystinuria patients have >300 mg per day. Genetic testing is not always required but could be useful in atypical cases [13].

Cystinuria commonly predisposes to recurrent episodes of urolithiasis, leading to renal insufficiency and chronic kidney disease if left untreated. Management includes both management of acute stone episodes, which in our case necessitated surgical procedures and prevention of disease recurrence. Concerning the prevention of stone formation, interventions to reduce cystine excretion and increase cystine solubility in urine are used. Dietary habits modification, including appropriate hydration with 1.5L/m2 body surface in children and restriction in sodium and protein consumption, are warranted [14]. In addition, pharmaceutical interventions that include the administration of sodium bicarbonate for urine alkalinization and tiopronin to control cystine precipitation and excretion might be required. However, despite the abovementioned therapeutic interventions, patients often have to undergo multiple surgical procedures to manage urolithiasis recurrence [15].

## Conclusions

This case report highlights the importance of clinical suspicion and thorough investigation for pediatric patients presenting with recurrent episodes of UTIs. Cystinuria is a rare cause of urolithiasis, typically presenting with an earlier onset of recurrent urinary tract infection episodes compared to other urolithiasis causes. Stone analysis remains the cornerstone for confirming cystinuria diagnosis. Recurrent or atypical urinary tract infections should raise clinical suspicion for potential underlying abnormalities, including CAKUT and urolithiasis. A stepwise diagnostic approach based on initial clinical and imaging studies is warranted.
